# A computational exploration of resilience and evolvability of protein–protein interaction networks

**DOI:** 10.1038/s42003-021-02867-8

**Published:** 2021-12-02

**Authors:** Brennan Klein, Ludvig Holmér, Keith M. Smith, Mackenzie M. Johnson, Anshuman Swain, Laura Stolp, Ashley I. Teufel, April S. Kleppe

**Affiliations:** 1 Network Science Institute, Northeastern University, Boston, MA USA; 2 Laboratory for the Modeling of Biological and Socio-Technical Systems, Northeastern University, Boston, MA USA; 3 Center for Data Analytics, Stockholm School of Economics, Stockholm, Sweden; 4 Department of Physics and Mathematics, Nottingham Trent University, Nottingham, UK; 5 Department of Integrative Biology, University of Texas at Austin, Austin, TX USA; 6 Department of Biology, University of Maryland, College Park, MD USA; 7 Graduate School of Science, University of Amsterdam, Amsterdam, The Netherlands; 8 Santa Fe Institute, Santa Fe, NM USA; 9 Texas A&M University, San Antonio, San Antonio, TX USA; 10 Institute for Evolution and Biodiversity, University of Münster, Münster, Germany; 11 Department of Clinical Medicine (MOMA), Aarhus University, Aarhus, Denmark

**Keywords:** Evolvability, Complexity

## Abstract

Protein–protein interaction (PPI) networks represent complex intra-cellular protein interactions, and the presence or absence of such interactions can lead to biological changes in an organism. Recent network-based approaches have shown that a phenotype’s PPI network’s *resilience* to environmental perturbations is related to its placement in the tree of life; though we still do not know how or why certain intra-cellular factors can bring about this resilience. Here, we explore the influence of gene expression and network properties on PPI networks’ resilience. We use publicly available data of PPIs for *E. coli*, *S. cerevisiae*, and *H. sapiens*, where we compute changes in network resilience as new nodes (proteins) are added to the networks under three node addition mechanisms—random, degree-based, and gene-expression-based attachments. By calculating the resilience of the resulting networks, we estimate the effectiveness of these node addition mechanisms. We demonstrate that adding nodes with gene-expression-based preferential attachment (as opposed to random or degree-based) preserves and can increase the original resilience of PPI network in all three species, regardless of gene expression distribution or network structure. These findings introduce a general notion of *prospective resilience*, which highlights the key role of network structures in understanding the evolvability of phenotypic traits.

## Abstract (web summary)

Brennan Klein et al. propose an adapted network resilience measure, the prospective resilience (PR), to explore the resilience of protein–protein interaction networks following addition of new nodes. They apply PR to public datasets and find that resilience of a network is maximized when attachment of new nodes, or proteins, is based on the gene expression levels of the existing proteins in the network.

## Introduction

Evolution by natural selection acts upon already existing genetic material. Alterations like genetic mutations can cause deleterious effects and are commonly selected against^1^. However, emergence of evolutionary novelty is needed for traits to evolve as the environment is constantly changing. Thus, an evolutionary balancing act is needed to acquire beneficial novelty and simultaneously avoid deleterious traits.

The evolutionary trajectory by which novel features may be incorporated into already existing molecular systems is not well understood. An extensive amount of research has been dedicated to our understanding of protein sequence evolution, and what may enable adaptation without disrupting already present biological functions. The functional divergence of genomes has been explored by studying gene duplication^2–4^, de novo gene emergence^5–10^, open reading frame extension^11–13^, and sequence properties^14,15^, i.e., GC-content^16^ and codon usage^17–19^. While there has been much focus on addressing wherefrom and how novel sequence features emerge (e.g., gene duplication, de novo gene emergence), limited attention has been given to how novelty may become integrated into the cellular apparatus from a systems-level perspective and what systems-level processes facilitate the incorporation of novel interactions.

Research of essential genes suggests that classification of *gene essentiality* is context dependant and quantitative rather than a static and qualitative feature^20^. In fact, what determines gene essentiality and gene dosage-sensitivity has been suggested to be dependant on genetic and cellular context, and in part reflected in biological networks^20–22^. Whether a novel protein is deleterious or beneficial depends not only on its own sequence features, but also the environmental context of available interaction partners^23–26^. It is, therefore, fundamental to understand how a protein interacts with its proteomic surrounding.

Here, we examine the resilience of protein–protein interaction (PPI) networks as the network changes. Biological resilience is a measure of how tolerant a system is to perturbations^27^. This notion of resilience is related to the system’s *redundancy*^28^; biological redundancy refers to two or more components performing equivalent functions in a given biological system, such that deactivation of one of them has negligible consequences on the performance of the biological phenotype. Previous research has shown that biological redundancy has a positive association to network connectivity^29,30^ and may enable biological resilience by increased tolerance to perturbations in PPI networks^31^. Here, a perturbation is defined as an alteration; either adding or removing a protein of a given network. Adding or removing a protein in a PPI network will alter the connectivity and therefore also the network resilience.

**Fig. 1:**
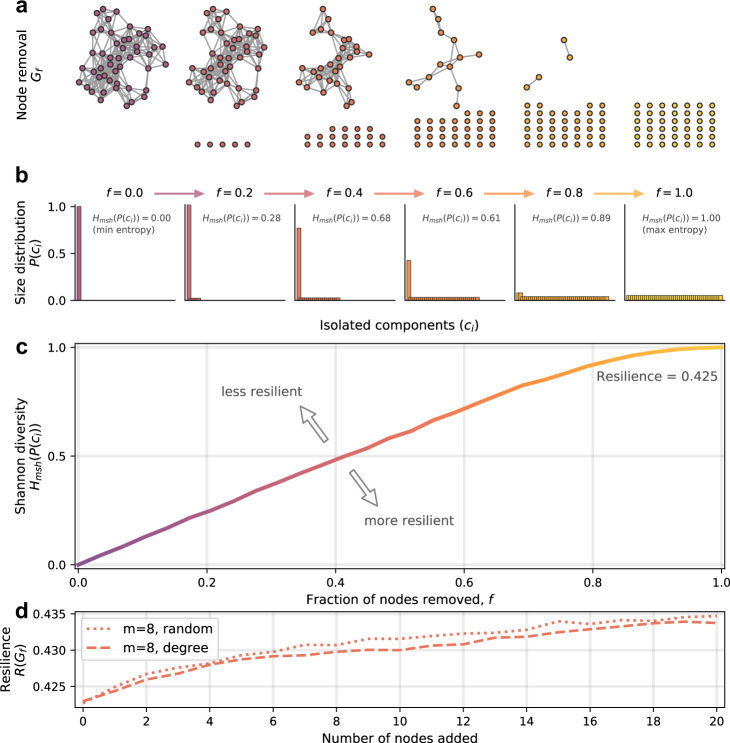
Change in the Shannon diversity and network resilience. A visual intuition is provided to depict how network structure is associated with a particular resilience value. **a** Network resilience is calculated by iteratively isolating fractions of nodes in the network, *f*, eventually leaving *N* isolated nodes. **b** Following every iteration, the Shannon diversity of the component size distribution is calculated, in this case starting at *f* = 0 (one connected component), and increasing until every node is disconnected, *f* = 1. **c** Increasing the fraction of nodes that have been isolated creates a curve of increasing entropy values, which is used to compute the network resilience, as in Eq. (2). **d** An example of the *prospective resilience* of the network shown in (**a**). New nodes are iteratively added to the original network, with *m* links attached randomly or preferentially based on the degree of nodes in the network.

“Network resilience”, as defined by Zitnik et al.^31^, describes the extent to which random node isolation deteriorates network structure (node isolation here being where all links are stripped from the node, leaving it isolated from the rest of the network, see Fig. 1a). Assuming that tolerance for novelty is linked to network resilience, we aim to analyse which features affect resilience and enable successful integration of novel proteins into PPI networks. Essentially, we are asking to what extent biology may be shaped by, or is making use of, the general properties of statistical network science relating to attachment mechanisms in the development of “resilient” protein interaction networks. To this end, we use network science to computationally explore how novel proteins may become integrated in PPIs. Specifically, we introduce and apply a novel network measure referred to as the *prospective resilience*. This involves introducing new proteins to a network based on different attachment rules and measuring the resulting network’s resilience compared to baseline. By measuring the change in network resilience following the addition of new nodes to the network, we are able to infer how robust a given network structure is to incorporating novel proteins. We examine the prospective resilience of PPI networks under three different mechanisms for attaching novel proteins to the network. These mechanisms include a random-attachment strategy, a degree-based attachment strategy common in the generation of many scale-free networks, and a biologically inspired gene expression-based attachment strategy, as it has been suggested that protein evolution and network topology are interlinked with protein abundance (gene expression)^32–36^.

**Fig. 2:**
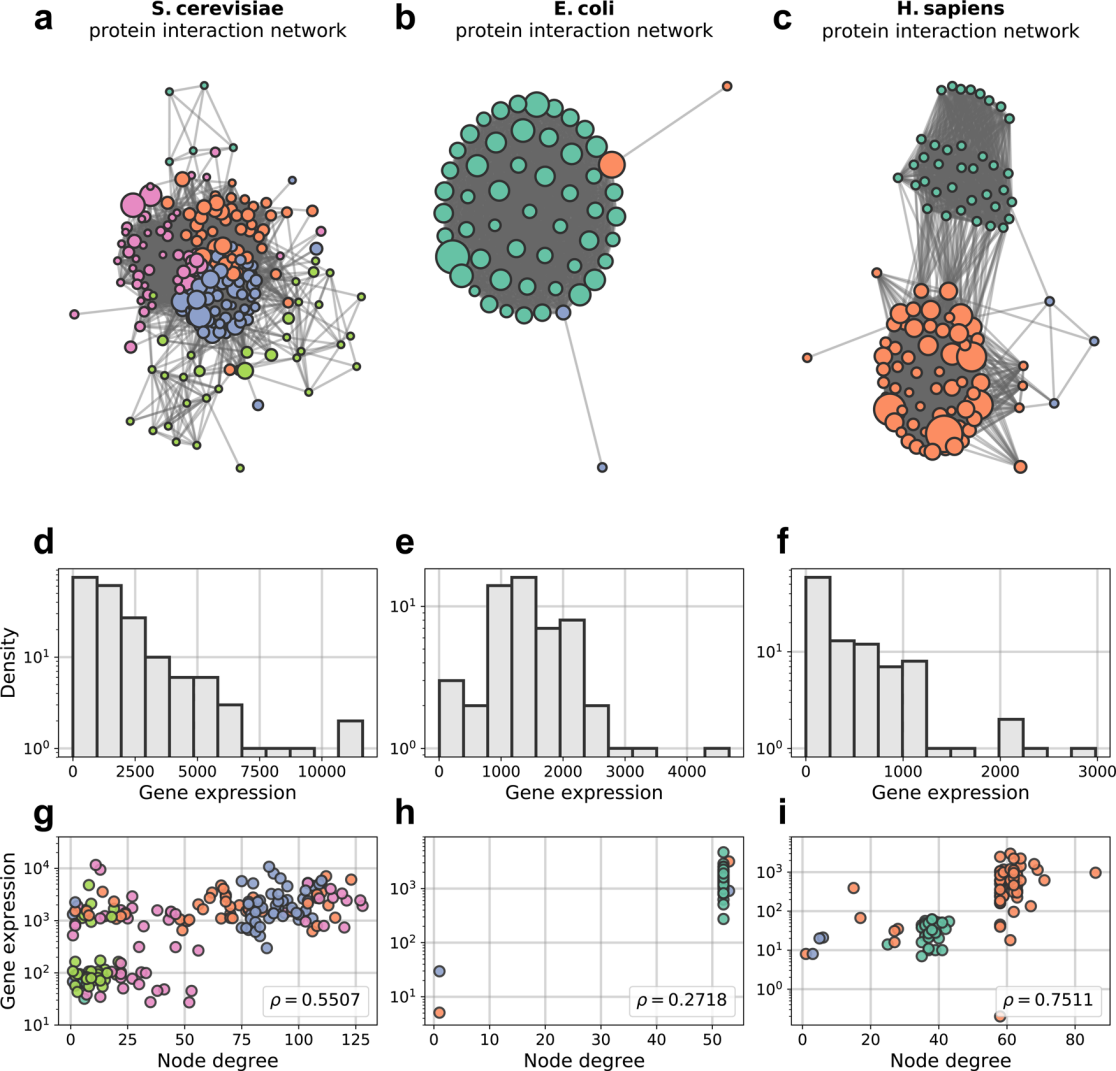
Ribosomal networks. These species have ribosomal interaction networks that span a range of different network structures. Node colors depict detected communities in the networks. Nodes of a given color are more likely to connect to other nodes of that color. Node size is proportional to gene expression. **a** *S. cerevisiae* ribosomal network. **b** *E. coli* ribsomoal network. **c** *H. sapiens* ribosomal network. **d**–**f** Gene expression distribution of ribosomal networks for *S. cerevisiae*, *E. coli*, and *H. sapiens* respectively. **g**–**i** Gene expression (in transcripts per million, TPM) plotted against node degree for (*S. cerevisiae*, *E. coli*, *H. sapiens*), respectively. To accentuate clusters of nodes that share degree and gene expression attributes, the points in these plots share the same color as their corresponding nodes in (**a**–**c**). Node size is not included here to improve clarity.

Here, we analyze PPI networks with available data with respect to gene expression, PPIs and network structure (see Fig. 2). We examine the PPI networks of DNA repair, mismatch repair, DNA replication, and the ribosome. We make use of publicly available data (SNAP^37^ and KEGG^38^ databases), which are annotated and experimentally verified, for three organisms: *Escherichia coli* (prokaryote), *Saccharomyces cerevisiae* (unicellular eukaryote) and *Homo sapiens* (multicellular eukaryote). We found that the prospective resilience of many of these networks is greater when node addition was based on the gene expression compared to the other node attachment strategies.

## Results

### Network resilience and prospective resilience

In biological terms, individual nodes represent individual proteins of the PPI network, and we infer the biological resilience by inferring network resilience. The network resilience, *R*, is an information theoretic measure that describes the extent to which random node isolation deteriorates network structure^31^. This deterioration is determined by the growing number of connected components in the network as links are removed. Recall, that a connected component in a network is a subset of nodes for which any two nodes are connected by at least one path and two nodes are in different connected components if no path exists between them in the network. It is computed iteratively, involving the incremental isolation of (i.e., removal of all links to) more and more nodes in the network. In biological terms, links represent protein interactions, and the removal of links represents the removal of an interaction between two proteins, yielding isolated and non-interacting proteins. The number of nodes isolated is the fraction $f=\frac{a}{b}$ of all nodes in the network (rounded to the nearest number of nodes), where *b* is the total number of iterations and *a* increases from 0 to *b* in steps of 1, i.e., if *b* = 100, we isolate 0%, 1%, 2%, ... 100% of the nodes. At each iteration, a modified Shannon diversity measure,

1 $${H}_{msh}(G)=-\frac{1}{{{{{{{\mathrm{log}}}}}}}\,(N)}\mathop{\sum }\limits_{x=1}^{X}{p}_{x}{{{{{{\mathrm{log}}}}}}}\,{p}_{x}$$

is computed for the resulting network, where ${p}_{x}=\frac{| {c}_{x}| }{N}$, *c*_*x*_ is a connected component of the network, and *N* is the number of nodes; *p*_*x*_, therefore, is the probability that a randomly-selected node is in the connected component *c*_*x*_. As *f* increases from 0 to 1, the network becomes more and more disconnected until *f* = 1, at which point the resulting network, *G*_*f* = 1_, is a collection of *N* isolated nodes (Fig. 1a, b). Consequently, the Shannon diversity of these component size distributions increases with *f* (Fig. 1c). The final value for resilience is then calculated as a discrete approximation of the area under this curve:

2 $$R(G)=1-\mathop{\sum }\limits_{a=0}^{b}\frac{{H}_{msh}({G}_{f = a/b})}{b}$$

where *H*_*msh*_(*G*_*f*_) is the modified Shannon diversity of the network after *f* fraction of nodes have been disconnected. In Supplementary Note S1, we break down the typical behavior of this resilience measure. Particularly, we show that dense Erdős-Rényi networks are more resilient than sparse ones (Supplementary Fig. S1), which conforms to the intuition that a complete network is the most resilient network, with a value *R*(*G*) = 0.5. Note that this measure was previously defined as ranging from 0.0 to 1.0^31^, but we show that the theoretical maximum is in fact 0.5 (see Supplementary Note S1).

Here, we introduce a novel adaptation of this resilience measure, which we refer to as the prospective resilience (*PR*). The intuition behind this measure is to ask to what extent the resilience of a given network changes following the addition of new nodes into the network structure. In a biological context, this models how a network responds to the introduction of new proteins. Building on common modeling techniques for studying network growth processes, the prospective resilience is obtained by repeatedly adding new nodes to the network and calculating the updated resilience of the resulting network. This yields a vector of resilience values, {*R*_*t*+1_(*G*), *R*_*t*+2_(*G*), . . . , *R*_*τ*_(*G*)}, corresponding to the resilience of the network after the addition of each of the *τ* new nodes to the network:

3 $$P{R}_{\tau }(G)={\left\{{R}_{t}(G)\right\}}_{t = 1}^{\tau }$$

**Fig. 3:**
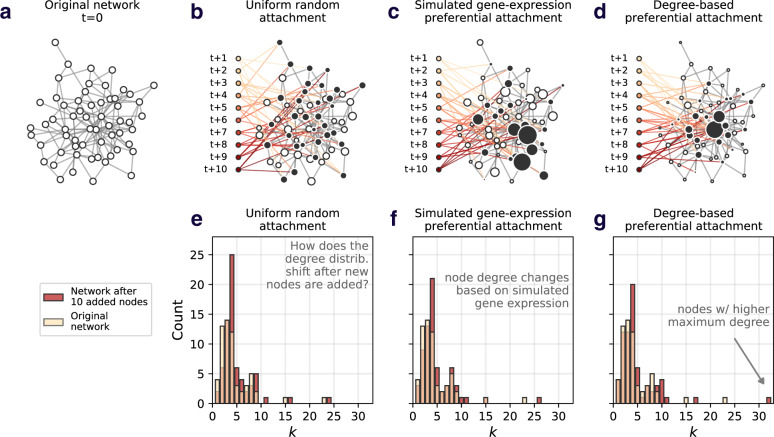
The effect of attachment mechanism on network structure. A visual depiction of the effect of adding nodes under different attachment mechanisms. In each example, 10 nodes are added, connecting their *m* = 4 links to nodes in the original network (indicated by the black nodes). Node size corresponds to its likelihood of gaining new links. **a** Example network, before node addition. **b** Example of uniform attachment. **c** Example of (simulated) gene expression preferential attachment. **d** Example of degree-based preferential attachment. **e**–**g** Depicts the change in the original network’s degree distribution after the addition of 10 nodes, under each attachment mechanism (uniform, gene expression, and degree based). The white bars are transparent to show overlap. While these histograms highlight the change in a single network property (degree, *k*), one can imagine a number of structural changes occurring following the addition of new nodes, depending on the attachment mechanism.

Given that the prospective resilience is computed by adding nodes to a network, the mechanism by which nodes are added becomes an important consideration. In general, node attachment mechanisms assign a probability that each incoming node, *v*_*t*+1_, attaches its *m* disconnected links (often referred to as “dangling” links) to nodes already in the network, *v*_*i*_ ∈ *V*. This could be based on *random* attachment, where each node, *v*_*i*_, has a uniform probability ${p}_{i}=\frac{1}{N}$ of becoming connected to the incoming node, *v*_*t*+1_. Similarly, a new node can add its *m* links preferentially based on the *degree* (number of neighbors) of the nodes in the network, _*pi*_ ∝ *k*_*i*_, where *k*_*i*_ is the degree of node *v*_*i*_. This means that the probability that *v*_*i*_ will receive an incoming link is ${p}_{i}=\frac{{k}_{i}}{2E}$, where *E* is the total number of links in the network. Figs. 3a–d show examples of different attachment mechanisms and how the different mechanisms change the structural properties of the original network (Fig. 3e–g).

From the biological perspective, we posited that a novel protein entering a system is inevitably more likely to interact with proteins that are more abundant in that system. This abundance can be determined by the protein’s gene expression^39,40^. To this end, we compare the random and degree-based attachment mechanisms with attachment based on gene expression. This is implemented exactly as for degree-based attachment; the probability that node *v*_*i*_ receives an incoming link is proportional to *v*_*i*_’s gene expression (i.e., the gene expression of node *v*_*i*_ divided by the sum of the gene expressions of all nodes). New nodes (novel proteins) will not have a known gene expression, and as such, we assign them the average gene expression of the network. Through this attachment rule, we explicitly couple insights from network science to the biological properties of protein networks.

### Protein–protein interaction networks

In this work, we explore the notion of prospective resilience in biological systems. To do so, we focus on PPI networks from three species: *S. cerevisiae*, *E. coli*, and *H. sapiens*. In this section, we introduce the procedure for generating these PPI networks.

Each protein in a species’ PPI network is represented by a node. The links between nodes were then established wherever there was evidence of PPIs in that species, based on data from the SNAP database^37,31^. We identified proteins belonging to respective PPI networks from data in ref. ^38^ and constructed the ribosomal protein networks based on data from the SNAP database^37^, which is a selected subset of the STRING database^41^. SNAP consists of physical PPIs that are curated by experimental verification. Note, the links in these networks are unweighted, indicating that either a PPI has been established between two proteins or has not, with no indication of strength of interaction included.

Expression for *S. cerevisiae* came from NCBI GEO^42,43^, *H. sapiens* from EMBL-EBI Expression Atlas^44,45^, and *E. coli* K12 from NCBI GEO^42,46^. See “Data sources” section for a detailed description on how the networks were constructed and how their associated gene expression data was collected. Visualisations of these networks are shown in Fig. 2a–c, and several network properties reported in Table 1. In Figs. 2d–f, distributions of gene expression for each network are plotted as histograms and against node degree. The distributions for all three species had heavy tails, with small numbers of highly expressed proteins and a bulk of proteins with relatively low expression. Across the three networks included here, we see that nodes with similar gene expression and degree tend to cluster together, however the correlation between degree and gene expression itself varies between species (Fig. 2g–i, with Spearman rank correlation coefficients included).

**Table 1:** Basic network measures.

Network property	*S. cerevisiae*	*E. coli*	*H. sapiens*
Network size	145	55	105
Density	0.284	0.929	0.471
Average degree	40.82	50.18	48.93
Resilience	0.438	0.435	0.444
Modularity	0.182	0.0013	0.363

Network size is number of nodes/proteins. Network density is the fraction of the actual amount of edges over the possible amount of edges. Average degree is the average number of edges per node. Resilience and modularity are described in further detail in section “Network modularity” and Supplementary Note S1.

#### Prospective resilience in protein–protein interaction networks

We computed prospective resilience under a number of different scenarios in order to determine the conditions under which networks would have the highest prospective resilience (i.e., which attachment mechanism is the most effective for maximizing the network’s prospective resilience). In each condition, we calculate the prospective resilience by adding 20 new nodes to each network. We varied the number of new links, *m*, that each new node added to the network (*m* = 4, 8, and 16). Each simulation was repeated 100 times and the means and standard deviations were recorded from these runs. The resilience was calculated with a rate of node isolation, *b* = 50 (see “Network resilience and prospective resilience” section).

**Fig. 4:**
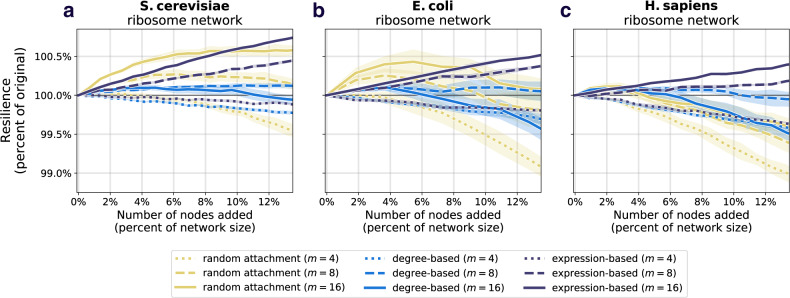
Prospective resilience of three ribosomal networks. As more nodes are added (horizontal axes), the resilience of the resulting network changes (vertical axes). The color of each curve corresponds to the number of new links that each new node enters the network with, and the line style (solid, dashed, or dotted) corresponds to the three different node attachment mechanisms. **a** Prospective resilience of *S. cerevisiae* ribosomal network. **b** Prospective resilience of *E. coli* ribosomal network. **c** Prospective resilience of *H. sapiens* ribosomal network. Ribbons around each curve correspond to their 95% confidence intervals.

The results comparing the prospective resilience across the three species and attachment mechanisms are shown in Figs. 4a–c and in Supplementary Figs. S3j–l, S4j–l, and S5j–l, for the ribosomal network, the DNA replication network, mismatch repair network, and the protein export network, respectively. We consistently found that the most effective mechanism for adding new nodes to the networks was the attachment rule based on the gene expression of nodes in the original network. See Supplementary Note S3 for supplementary results.

Degree-based and random attachment were on average less effective at increasing the resilience of these networks (though there is a slight improvement in *S. cerevisiae* in the case of random attachment, a trend that disappears as more nodes are added). In general, a higher positive slope indicated that the attachment rule (along with the number of links that each new node enters the network with) generated higher prospective resilience. For information about the statistical differences between the slopes of each curve in Fig. 4, see “Statistics of prospective resilience and modularity” section and Supplementary Note S2. Note, it is observable that the confidence intervals for the gene-expression mechanism tended to be tighter than for random attachment and degree distribution. One straightforward explanation for this is that the heavy tail of the gene expression distribution (as compared to degree distribution and uniform distribution associated with random attachment) would create more similar patterns of attachment for newly added nodes in the network, i.e., in each iteration being more likely to attach to the same high gene expression nodes, thus more predictable results in the prospective resilience analysis.

In order to put these results in a better context, we performed a survey of resilience in random networks as the inference of network resilience has been under-explored for random networks. In Supplementary Note S1.1, we include several explanatory simulations that offer a more comprehensive intuition about how this measure behaves in networks. We highlight two main behaviors of this measure: its dependence on the network density and the *degree heterogeneity* of the network. We illustrated this further in the context of Erdős-Rényi networks and preferential attachment networks (Supplementary Notes S1.1 and S1.2).

Based on our analyses of random networks, adding more links (therefore making the resulting network more dense) increased the prospective resilience in each of the three networks. This is shown by the different colored lines in Fig. 4, as well as in Supplementary Figs. S3–S5. This holds regardless of the method of attachment. In other words, given that *links* in these networks correspond to interactions between proteins, our results suggest that a network’s resilience is more likely to increase if novel proteins are highly interactive and particularly if they are highly interactive with highly expressed proteins that are already present in the network. Through these results with random networks, as well as our additional analyses on several PPI networks (Supplementary Figs. S3–S5), our findings suggest that there is a key role that the interplay between network structure and gene expression has for determining a network’s structural resilience. As the results regarding resilience appear independent of what PPI network one analyses, we chose to focus on just one PPI network for the remaining analyses (modularity and noise) as representative for all of them; the ribosomal PPI network. The ribosome is the biggest of these PPI networks, in addition of being curated by extensive previous research^47–50^, giving it the strongest statistical power and reliability.

#### Resilience and modularity

We found that the gene expression-based attachment mechanism was most effective at maximizing the prospective resilience of the three networks included here. This finding does not immediately account for the extent to which this could have been due to higher-order, structural (i.e., not necessarily biological) properties of the network measured by classical network metrics. Particularly, the networks showed observably strong community structure, a property that can be measured by some metric for modularity. We, therefore, tested whether the observed results could be explained more straightforwardly by modularity using the common modularity metric proposed by Newman and Girvan^51^:

4 $$Q=\frac{1}{2m}\mathop{\sum}\limits_{i,j}\left[{A}_{ij}-\frac{{k}_{i}{k}_{j}}{2m}\right]\delta ({c}_{i},{c}_{j}),$$

where *m* is the number of edges, *A*_*ij*_ is the element of the adjacency matrix in row *i* and column *j*, *k*_*i*_ is the degree of *i*, *c*_*i*_ is the module assigned to node *i*, and *δ*(*x*, *y*) is the Kronecker delta function which is 1 if *x* = *y* and 0 otherwise.

In general, we refer to networks as being modular when they consist of densely-connected clusters of nodes that connect more to each other than to the rest of the network. We chose to analyse modularity due to observations of strong modular structures in all of the networks, especially in the case of *H. sapiens* (Fig. 2c). Additionally, we note that the three networks have very different initial levels of modularity (Table 1).

**Fig. 5:**
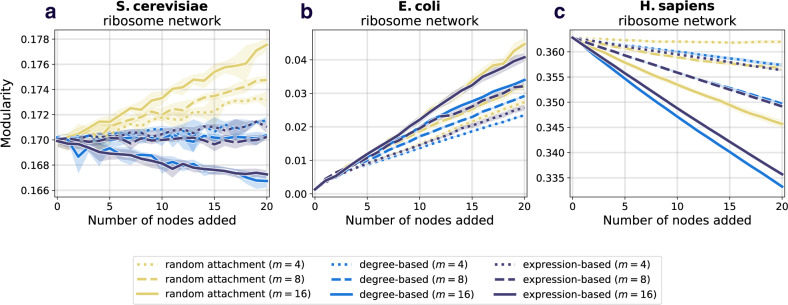
Prospective modularity of three ribosomal networks. As a comparison measure, we also examine how the modularity of the network changes following the addition of new nodes. The color scheme and line styles are the same as in Fig. 4. **a** Prospective modularity of *S. cerevisiae* ribosomal network. **b** Prospective modularity of *E. coli* ribosomal network. **c** Prospective modularity of *H. sapiens* ribosomal network. Crucially, we do not find any evidence that the prospective resilience results observed in Fig. 4 are being driven by the change in the networks' community structures, as the plots here show highly divergent patterns, suggesting that there is a more distinct mechanism underlying prospective resilience.

Here, we examine whether we observe similar results to those in section “Prospective resilience in protein–protein interaction networks“ if we instead look at the change in the networks’ modularity following the introduction of new nodes. To do this, we computed the modularity of the network after each addition of new nodes. Full details of the analysis are found in section “Network modularity”. We found that the behavior of prospective modularity did not resemble the observed trends for prospective resilience (Fig. 5). In fact, node addition affected the prospective modularity of each network differently, with no discernible pattern between the different networks. As such, modularity was ruled out as an explanatory measure for network resilience. In conclusion, the modular structure of the networks included here did not drive their prospective resilience.

#### Noise and protein networks

We previously observed that gene expression was moderately correlated with node degree while gene expression-based attachment performed better than degree-based attachment. Here, we examine how decoupling of gene expression from the network topology affects the prospective resilience of the network. In other words, we probe to what extent the performance of gene expression-based attachment is influenced by the distribution (i.e., Figs. 2d–f) of gene expression values and its potential to create novel network structure, rather than any relationship between the gene expression values and the PPI network’s existing topology. To do this, we randomly shuffled the gene expression values across the network and re-ran the prospective resilience simulations. We did this for different amounts of shuffling. For example, at 20% shuffling, the gene expression values for a randomly chosen 20% of the proteins (network nodes) were subject to a random permutation, while the remaining 80% of proteins retained their original gene expression. At 100% noise the gene expression values were randomly assigned to nodes across the network.

**Fig. 6:**
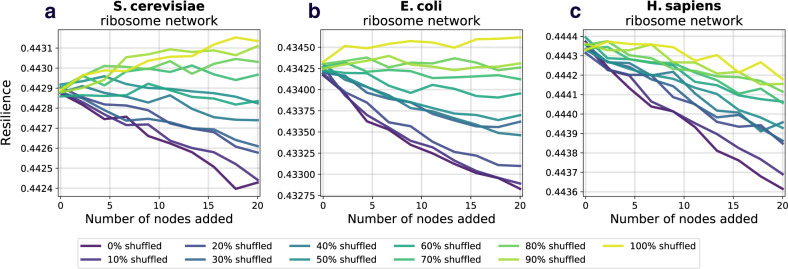
Prospective resilience and randomized gene expression. We examine if specific gene expression is driving the high prospective resilience of the expression-based attachment rule or if merely attaching nodes based on a shuffled gene expression distribution could bring about these results. Each new node joins with *m* = 5 for *S. cerevisiae* and *E. coli*, and *m* = 6 for *H. sapiens*. These values were selected so that the slope of the prospective resilience would be closest to 0.0 when the gene expression was not shuffled (0% shuffled). See Table 2 for how the correlation between a node’s degree and its gene expression changes as noise increases. **a** Prospective resilience of *S. cerevisiae* ribosomal network. **b** Prospective resilience of *E. coli* ribosomal network. **c** Prospective resilience of *H. sapiens* ribosomal network. Notably, we find that the prospective resilience of the networks *increases* simply by increasing the fraction of nodes with shuffled gene expressions.

We observe, in each of the three networks, that elevated shuffling of gene expression *increased* prospective resilience (Figs. 6a–c). In other words, biological noise simulated as random distribution of expression, increases prospective resilience. It makes sense that some noise would increase the prospective resilience; resilience increases as networks becomes more dense, and shuffling the gene expression values may increase the chance that a given low-degree node receives a link from an incoming node. However, increasing noise always increased the prospective resilience. This can be explained by the fact that the simulations reported here do not consider the biological limitations that a real protein interaction network would face (e.g., gene dosage imbalance); our simulations only address the resilience of the network structures.

Therefore, we conclude that the effect of the uneven distribution of gene expression (and its limited association with degree) on the preferential attachment mechanism promotes new hubs (higher degree nodes) of connectivity in the network, which increases the network’s prospective resilience. The greater the novelty in the network structure created by this mechanism (i.e., the less correlation between degree and gene expression) the greater the network’s prospective resilience (Table 2).

**Table 2:** Spearman rank correlation, *ρ*, between the degree and gene expression of a network at different levels of noise.

	*S. cerevisia*		*E. coli*		*H. sapiens*	
Noise	*ρ*	*p*-value	*ρ*	*p*-value	*ρ*	*p*-value
0.0%	0.55	1.06e^−16^	0.27	4.47e^−02^	0.75	2.78e^−20^
20.0%	0.44	1.03e^−10^	0.23	4.47e^−02^	0.61	4.04e^−12^
40.0%	0.33	2.18e^−06^	0.16	1.72e^−01^	0.45	1.23e^−06^
60.0%	0.22	1.84e^−03^	0.11	3.07e^−01^	0.31	1.47e^−03^
80.0%	0.11	1.22e^−01^	0.06	4.04e^−01^	0.15	1.21e^−01^
100.0%	−0.0	5.17e^−01^	−0.0	4.78e^−01^	-0.0	4.95e^−01^

The table displays the correlation after *Noise %* has been introduced to the network. The Spearman correlation was run over the mean from 1000 iterations.

## Discussion

This study used new network scientific methods to undertake a systems approach to understanding how novelty is incorporated into protein–protein interaction (PPI) networks. We accomplished this by adapting a measure of *network resilience* to characterize the *prospective resilience* of multiple PPI networks. We found that the prospective resilience of the many of the networks examined was greatest when node addition was based on the gene expression of the proteins in the original networks. This suggests that the distributed levels of gene expression among proteins facilitates or enables the system of interacting proteins to receive and incorporate new proteins. It also suggests an important correspondence between the structure and biological properties of protein networks.

We also undertook a survey of how network resilience behaves in random and preferential attachment networks, and highlighted its dependence on the density and degree heterogeneity of the network (see Supplementary Note S1). These simulations contextualize the analyses that we performed for ribosomal networks and provide a platform for further use of the metric in a more theoretical sense.

We compared the prospective resilience to a meso-scale network structural measure (which we refer to as the *prospective modularity*) to determine if the observed increases in resilience were due to the more widely studied property of community structure^51^. No clear trend between prospective resilience and prospective modularity was found between the networks (Fig. 5). This supports the hypothesis that there remains a crucial role of gene expression specifically in the resilience of a PPI network.

In a biological setting, network resilience infers biological redundancy. We assume that novel proteins can be integrated into existing PPI networks if they do not cause the network to become disconnected, and instead add to the network redundancy. We find that likelihood of a novel protein being integrated is dependent on the existing topology of PPI and internal connectivity, but also gene expression. The results of our node attachment analysis imply that novel proteins are able to be integrated if they (i) are interactive with many existing proteins, or (ii) primarily interact with proteins that are more abundant (inferred by gene expression)^52^.

We also found that shuffling gene expression tends to further increase resilience. The heavy tails of the gene expression distributions may indicate that (i) the most important factor for increasing resilience is the creation of new hubs of connectivity (new nodes strongly connecting to a few existing nodes), and (ii) these new hubs are more effective in increasing resilience if created randomly in the network and not correlated with the already established topology. Interestingly, a heavy-tailed (log-normal) factor of attachment has been recently demonstrated as an accurate explanation of the degree distributions across various complex networks^53^, lending credence to the idea of gene expression as (at least part of) such an explanatory mechanism in PPI networks. If gene expression influences the evolution of the PPI networks, then it necessarily needs to have an amount of correlation with the existing degree distribution of the network. Thus, even though we observe that the completely randomised gene expression across the network yields a more resilient network, given enough time, the network connectivity would evolve to correlate with the new gene expression values of the corresponding proteins. Then, more noise would be required to increase the network resilience.

In an evolutionary trajectory of a PPI network, we would thus expect to see a trade-off between the topological influence of gene expression (i.e., correlation between gene expression and protein node degree) and the emergence of novelty through biological noise (i.e., weakened correlation between node degree and gene expression). Arguably, this is reflected in the weak to moderately strong correlations found in Fig. 2g–i. This conforms to classic theoretical notions of the usefulness of noise in biological systems^54,55^. In light of research in population genetics, species with small effective population size are observed to undergo a higher mutation rate due to imperfect selective constraint^1^. In fact, it has been suggested that weakly deleterious mutations induce secondary selection for stabilizing protein–protein interactions and that biological complexity is a side-effect of non-adaptive processes^21,56^. Accordingly, species with small effective population size (e.g., multicellular eukaryotes) should have a higher interactome resilience and complexity due to higher exposure to noise, whereas species with large effective population size (e.g., bacteria) should have a smaller and less resilient interactome. This was observed by Zitnik et al.^31^ who studied resilience of species interactomes; vertebrae and other multicellular eukaryotes display a higher interactome resilience than unicellular eukaryotes and bacteria do^31^. However, whether interactome resilience is a feature selected for per se rather than a consequence of induced biological noise is ambiguous^57,58^. Further research is needed to establish to what degree noise is a contributing factor to PPI network resilience. Ultimately, resilience is not the only factor to consider in PPI network evolution, but it is informative of how well the PPI network may tolerate perturbations (e.g., mutations).

Our findings suggest that novel proteins might enter PPI networks and interact broadly as generalists. Previous research suggests how many proteins, i.e., enzymes, begin as generalists with many interacting partners, and later evolve more specialized interactions^52,59^, whereas ribosomal proteins may have evolved toward multiple functions while primarily acting as stabilizers of rRNA^60^. Indeed, our results seem to corroborate the “constructive neutral evolution”^61^, in that new nodes added to the network may not initially affect the resilience but over time contribute to the network’s complexity. Under this interpretation, novel proteins may be initially conserved in the network, simply by being tolerated and adding to the network resilience, as suggested in research on de novo genes^62^.

A recent phylogenetic inference of the evolutionary trajectory of the ribosomal PPI network—from bacteria to eukarya—found that novel interactions reinforced existing links or connected previously unconnected nodes^60^. The study did not report on network density, but suggested evidence toward increased network connectivity over evolutionary time contingent on emerging C-terminal sequence extensions next to globular domains. Previous research of protein evolution found protein substitutions to be contingent on prior epistatic substitutions, next to other sequence factors^63,64^. Taken together, it is worthwhile to explore the role of contingency, network resilience, noise, and gene expression combined when analysing the evolutionary trajectories of PPI networks.

Subsequent and systematic analyses of the prospective resilience of other species’ ribosomal networks (not to mention gene pathway networks, metabolic networks, etc.) will allow researchers to form more precise hypotheses about other possible mechanisms—especially ones relating gene expression, pairwise protein interactions and overall PPI network topology—which might be driving the results we observe and delineate here. In addition, it would be useful to explore how prospective resilience changes under other biologically-informed methods for introducing proteins into PPI networks, as well as networks with weighted connections between proteins. For example, the network connections used here indicate presence or absence of interaction, but there are circumstances where the measured interaction strength between proteins could be used to define *weighted* network connections. Novel proteins, e.g., duplicates of existing proteins, may have their attachment probabilities formed based on the interaction strength that the original protein has with other proteins. Additionally, as is the case in ribosomal complexes, proteins also interact with mRNA or other molecules not typically included in PPI data. Ultimately, we view this work as a first step toward understanding the stability of a network’s resilience to novel information and as such, we examined unweighted networks to highlight the importance of the presence or absence of connections in a network. Prospective resilience is a measure that can describe networks in general; it is particularly meaningful in the study of biological systems, but since complex systems are often described as recapitulating common properties across different domains, this network measure can be used in any system that undergoes and incorporates novel information.

## Methods

### Data sources

We make use of publicly available data of protein interaction networks from Zitnik et al. Full interactomes were obtained from their website (SNAP) for 3 model organisms: *Saccharomyces cerevisiae*, *Homo sapiens*, and *Escherichia coli* str. K12^37^. According to the documentation about the SNAP dataset, “In this study, however, we specifically focus on physical interactions and thus we exclude functional (indirect) associations from the analysis. We combine the following protein–protein interaction data: (a) Experimentally supported interactions... and (b) Human expert-curated interactions.”^31,37^.

We additionally gathered gene expression data for each of the species studied. Expression data for *S. cerevisiae* came from the wildtype data accessible on the NCBI GEO database (accession: GSE52119)^42,43^. The GTEx Consortium^45^ collected *H. sapiens* gene expression data for various tissues, which was accessed via the EMBL-EBI Expression Atlas^44^. We utilized expression reported in the spleen as it was the tissue where most of the genes in the ribosomal network were expressed. Gene expression for *S. cerevisiae* and *H. sapiens* was reported in transcripts per million (TPM) by original sources. Wildtype gene expression data for *Escherichia coli* str. K12 substr. MG1655 (NCBI:txid511145) was obtained from the NCBI GEO database (accession: GSE48829)^42,46^. Meysman et al. originally reported expression as count data; we converted from counts to transcripts per million (TPM) with custom R scripts and gene lengths for *Escherichia coli* str. K12 retrieved from UniProt^65^ in June 2019. To convert to TPM, we first divided the read counts by the length of each gene (in kilobases) to get reads per kilobase (RPK). The sum of all RPK values was divided by one million to produce a scaling factor, which was then multiplied by each protein’s RPK to produce their expression in TPM.

### Network resilience

A network, *G*, consists of *N* nodes, *V* = {*v*_1_, *v*_2_, …, *v*_*N*_}, connected by *M* links, *E* = {(*v*_*i*_, *v*_*j*_): *v*_*i*_, *v*_*j*_ ∈ *V*}. The resilience of a network is based on an information theoretic analysis of the distribution of the sizes of connected components in *G*^31^. A connected component may be defined as follows. If there exists a path of links between two nodes, *v*_*i*_ and *v*_*j*_, in *G*, then they are in the same connected component, *c*_*x*_, of *G*. Otherwise *v*_*i*_ and *v*_*j*_ are in separate components, *c*_*x*_ and *c*_*y*_, say, of *G*. If *v*_*i*_ has no links, and thus no paths from itself to any other node in *G*, then *v*_*i*_ is an isolated component of *G*. From this, we see that *G* is composed of *X* disjoint connected components, ${\{{c}_{x}\}}_{x = 1}^{X}$, of varying sizes such that $\mathop{\sum }\nolimits_{x = 1}^{X}| {c}_{x}| =N$. We can then confer a notion of probability to each component proportional to its size, *p*_*x*_ = ∣*c*_*x*_∣/*N*, such that if we chose a node at random from *G* it would have probability *p*_*x*_ of coming from component *c*_*x*_. Resilience is then measured through a modified Shannon diversity of the connected component size distribution in the presence of node isolation^31^, as follows:

5 $$H({G}_{f})=-\frac{1}{{{{{{{\mathrm{log}}}}}}}\,(N)}\mathop{\sum }\limits_{x=1}^{X}{p}_{x}{{{{{{\mathrm{log}}}}}}}\,{p}_{x}$$

This value is minimal, *H*(*G*_*f*_) = 0, when the network consists of a single connected component where paths exist between all node pairs, since ${{{{{{\mathrm{log}}}}}}}\,1=0$, and maximal, *H*(*G*_*f*_) = 1, when the network consists only of isolated components—$H(G)=-{{{{{{\mathrm{log}}}}}}}\,({N}^{-1})/{{{{{{\mathrm{log}}}}}}}\,N=1$. Through simulating the removal of a fraction of randomly-selected nodes, *f*, in a given network by removing all links to those nodes and leaving them as isolated components, we are left with a new network, *G*_*f*_. Then the entropy of the connected component distribution will increase with increasing *f*. With an increasing fraction of randomly isolated nodes, *f*, the entropy of the number of connected components will increase until *f* = 1.0, at which point there are *N* disconnected nodes (isolated components), reducing the network to the maximal case of *H*, as previously noted. We show an example of this process, as *f* increases, for an arbitrary simulated network (Figs. 1a–d). The resilience, *R*(*G*) of a network, *G*, is then defined as follows:

6 $$R(G)=1-\mathop{\sum }\limits_{f=0}^{1}\frac{H({G}_{f})}{{r}_{f}}$$

where *r*_*f*_ is the rate of node isolation such that $f\in \left\{\frac{0}{{r}_{f}},\frac{1}{{r}_{f}},\frac{2}{{r}_{f}},...,\frac{{r}_{f}}{{r}_{f}}\right\}$. In this work, we default to a value of *r*_*f*_ = 100, which means that the calculation of a network’s resilience involves iteratively isolating 0%, 1%, 2%, ..., 100% of the nodes in the network. For each value of *f*, we simulate the node isolation process 20 times.

### Structural modularity measure

#### Network modularity

Networks are often analyzed by their community structure—that is, to what extent do nodes in a network connect to other similar nodes, whether in their structural properties or specific attributes^51,66–68^. There are a number of different ways to detect community structure in networks, from algorithmic optimization to statistical/inferential to dynamical approaches^66,69,70^ (e.g., the color of the nodes in the networks in Fig. 2a–c was determined by one such approach^68^). Regardless of the community detection approach, each method outputs a partition that maps each node to a given community. The *modularity* of a given partition is a number that scores the extent to which it captures nodes’ tendencies to connect to other nodes in their same community at the expense of nodes in other communities^51^. While imperfect, this measure endows us with a powerful intuition for assessing higher-order network properties; namely, a network with high modularity partitions is likely to have obvious clusters of nodes, structurally separated from other parts of the network.

#### Prospective modularity

Here, we use the notion of modularity in an attempt to give possible explanations for the network mechanisms behind the observed trends in the prospective resilience of the ribosomal networks studied in this work. In particular, we define *prospective modularity* in the same vein as our prospective resilience measure to compare how node addition impacts resilience and modularity. The prospective modularity (*PM*) of a network is defined as the change in modularity following the addition of new nodes to a network (note the precise similarities between this measure and the prospective resilience). The addition of a new node, *v*_*t*+1_ with *m* disconnected links, to a network, *G*_*t*_, at time, *t* + 1 will likely change the modularity of the network. More specifically, by re-running a community detection algorithm on the resulting network, *G*_*t*+1_, and calculating the modularity of the resulting partition, we can observe the stability of this partition over time and ask whether the modularity will increase or decrease. Further, by varying the node-addition mechanism (adding nodes randomly, preferentially based on degree, or preferentially based on gene expression), we can observe the different effects that network structure and gene expression has on the prospective modularity of a given network.

### Statistics of prospective resilience and modularity

In order to determine the extent to which the curves in Fig. 4 differ from one another, we perform a series of statistical tests. The curves represent the average of 10 independent simulations for each condition. We utilize all existing simulation data here. For each value of *m* in each species, we perform an ANCOVA for each pair of attachment methods. We do a Bonferri-correction to correct for multiple testing and obtain a significance cutoff at *p* = 0.0166. Additionally, we calculate Cohen’s *d* from the *F*-statistic presented by the ANCOVA. The *p*-values and effect size (Cohen’s *d*) for each comparison are presented in Supplementary Table S1. Almost all of these slope comparisons are statistically significant. We do the same pairwise ANCOVA and effect size comparisons for the curves in Fig. 5 and report the outputs in Supplementary Table S2. For *S. cerevisiae*, *E. coli*, and *H. sapiens*, the majority of slopes are significantly different and show significant differences for larger values of *m*.

### Reporting summary

Further information on research design is available in the Nature Research Reporting Summary linked to this article.

## Supplementary information

Supplementary Information

Description of Additional Supplementary Files

Supplementary Data 1

Reporting summary

## Data Availability

The data used in this work are available at https://github.com/jkbren/presilience^71^ and in Supplementary Data 1. Supplementary Data 1 is a .json file that includes data for reproducing Figs. 4–6. Network data for recreating Fig. 2 is found at https://github.com/jkbren/presilience, and is stored as .graphml files in the /data folder; G_eco.graphml is the *E. coli* network, G_hsa.graphml is the *Homo sapiens* network, and G_sce.graphml is the *S. cerevisiae* network. Figs. 1 and 3 are generated from simulations, which can also be found at https://github.com/jkbren/presilience.

## Code availability

Software and reproducibility materials—including Python code with examples—can be found at https://github.com/jkbren/presilience^71^.
