# Supplementary material for: A computational exploration of resilience and evolvability of protein–protein interaction networks
Source: Commun Biol. 2021 Dec 2;4:1352. doi: 10.1038/s42003-021-02867-8 (PMC8639913; doi:10.1038/s42003-021-02867-8)
Supplement: Supplementary file 1 — Supplementary Information [file 42003_2021_2867_MOESM1_ESM.pdf]

Supplementary Notes:
A computational exploration of resilience and evolvability of protein-protein interaction networks

Brennan Klein, Ludvig Holmér, Keith M. Smith, Mackenzie M. Johnson, Anshuman Swain, Laura Stolp, Ashley I. Teufel, and April S. Kleppe

This supplementary information consists of theoretical and experimental work on the general properties of network resilience (Supplementary Note S1), and supplementary Tables S1 and S2 on statistical results on differences of the network prospective resilience and modularity curves for different attachment mechanisms.

Supplementary Note S1

Here, we describe resilience as calculated on two different random network models. One set of networks is generated by the Erdős-Rényi model and the other set is composed of preferential attachment models. Calculating the resilience of these networks offers insight into the behaviour of this resilience measure. In addition, we show here that the theoretical upper bound of the resilience measure is 0.5.

Supplementary Note S1.1

Resilience in Erdős-Rényi networks. In Erdős-Rényi networks nodes are connected uniformly at random. That is, each new node has a probability p to connect to one of the N nodes present in the network. In this way, the parameter p dictates the density of the network since a higher p means that each new node is more likely to form more edges (more of the possible edges between nodes that can exist, exists). Plotting resilience against p will then show us something about the relationship between resilience and density. Indeed, we observe a positive relationship between density and resilience (Figure S1 right). For a network with density close to 0 (i.e., low value of p) the resilience is also close to 0 and conversely a complete graph $p = 1$ yields a high resilience.

Supplementary Note S1.2

Resilience in preferential attachment networks. Preferential attachment networks are generated by the addition of new nodes, each with m dangling links (or disconnected links) [1, 2]. These m links connect to nodes, v_j, that are already present in the network

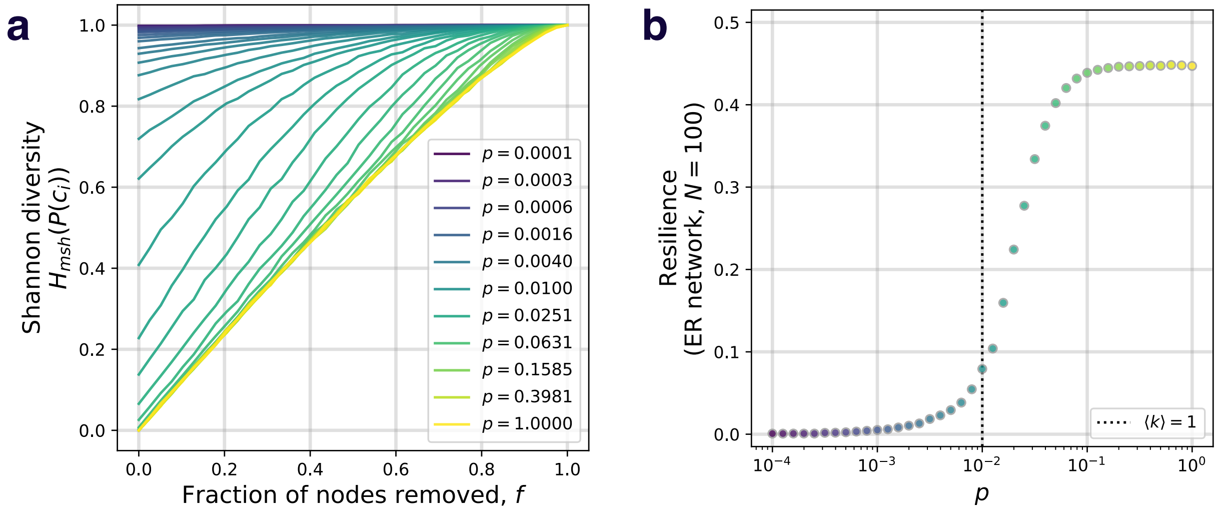

Figure S1: Erdős-Rényi and resilience. **(a)** Shannon Diversity changes as nodes are isolated from random attachment networks of 100 nodes for different values of p. For $p < 0.4$, the networks tend to have disconnected components before any of the nodes are isolated. This means that the Shannon diversity is greater than zero before any of the nodes are isolated. **(b)** The resilience values for the preferential attachment networks corresponding to panel **(a)**.

based on a probability proportional to k_j^α. Where k_j is the degree of node v_j and α a parameter which gives the amount of preferential attachment. $\alpha = 0$ means that there is no preferential attachment and the probability of attaching a dangling link to a node is the same for all nodes. $\alpha > 0$ means that dangling links are more likely to attach to nodes that have a high degree (nodes that already have a lot of links attached to them) and means that there is positive preferential attachment. Finally, $\alpha < 0$ means that dangling links are more likely to attach to nodes that have a low degree.

We observed that the resilience of a preferential attachment network depended on both α (Figures S2b, S2d, & S2f) and m (which varies between rows of subplots in Figure S2). Looking at the network where the number of dangling links, m, is 1, (Figure 2b), we see that resilience varies to a large extent as the tuning parameter α varies (roughly between 0.19 and 0.32). As m increases, the relationship between α and resilience changes and now low values of α yield higher values of resilience. In addition the spread of resilience decreases drastically, already for $m = 2$ resilience varies roughly only between 0.38 and 0.39 for different values of α. For $m = 24$, resilience hardly varies and there is no clear relationship between α and resilience. A higher m means that the networks are more dense. Therefore it seems that for dense networks, the structure of the network (governed by α) plays a less important role.

Supplementary Note S1.3

Upper bound for network resilience. It was originally claimed that resilience takes values in $[0, 1]$ [3]. However, we show that the upper bound of resilience is not higher than 0.5 as this is achieved asymptotically by the continuous counterpart of resilience applied to the complete graph of size N as $N \to \infty$.

The maximum resilience for a network of size N is achieved if, when all the links adjacent to any k nodes are removed, we end up with those k nodes as isolated nodes and the rest of the network remains connected as one component of size $N-k$, for all k. A network which satisfies this property is the complete graph, K_N. Take away the links of any k nodes in the complete graph and we have k isolated nodes and a complete subgraph of size $N-k$. In this instance, the modified Shannon diversity is:

$$\begin{aligned} H_k(K_N) &= -\frac{1}{\log N}\sum_{i=1}^{N} p_x \log p_x \\ &= -\frac{N-k}{N\log N}\log\left(\frac{N-k}{N}\right) - \frac{k}{N\log N}\log\left(\frac{1}{N}\right). \end{aligned}$$

Then, taking the above as a continuous function, integrating between 0 and N with respect to k and dividing by N (i.e. taking the average of the function) we get:

$$\begin{aligned} &\frac{1}{N}\int_0^N H_k(K_N)dk = -\frac{1}{N}\int_0^N \frac{N-k}{N\log N}\log\left(\frac{N-k}{N}\right) + \frac{k}{N\log N}\log\left(\frac{1}{N}\right)dk \\ &= -\frac{1}{N^2\log N}\int_0^N N\log\left(\frac{N-k}{N}\right) - k\log\left(\frac{N-k}{N}\right) + k\log\left(\frac{1}{N}\right)dk \\ &= -\frac{1}{N^2\log N}\int_0^N N\log(N-k) - N\log N - k\log(N-k)dk \\ &= -\frac{1}{N^2\log N}\left(\left[-N(N-k)\log(N-k) + N^2 - kN - kN\log N - \frac{1}{2}k^2\log(N-k)\right]_0^N - \frac{1}{2}\int_0^N \frac{k^2}{N-k}dk\right) \\ &=^* -\frac{1}{N^2\log N}\left[-\left(N(N-k)+\frac{1}{2}k^2\right)\log(N-k) + N^2 - kN(1+\log N) + \frac{1}{4}k^2 + \frac{1}{2}kN + \frac{1}{2}N^2\log(N-k)\right]_0^N \\ &= -\frac{1}{N^2\log N}\left(-N^2\log N + \frac{1}{4}N^2 + \frac{1}{2}N^2 + N^2\log N - N^2 - \frac{1}{2}N^2\log N\right) \\ &= \frac{1}{4\log N} + \frac{1}{2} \to \frac{1}{2} \text{ as } N \to \infty \end{aligned}$$

where * is achieved by polynomial division and then integration of the remaining integrand. Also, this is a decreasing function with respect to N, so $\frac{1}{2}$ is the minimum of this integral with respect to N, so an upper bound of the continuous resilience function, $R_c(G)$ is given by $1 - \frac{1}{2} = \frac{1}{2}$. Since the discrete function, $R(G)$ takes values equally spaced on $R_c(G)$ and $R_c(G)$ is a concave function, $\frac{1}{2}$ is also an upper bound for $R(G)$.

Species	m	Pairwise comparison	p-value	Cohen's d
S. cerevisiae	4	expression-based, degree-based	$4.86e^{-16}$	1.31
S. cerevisiae	4	expression-based, random attachment	$4.02e^{-11}$	0.89
S. cerevisiae	4	degree-based, random attachment	$1.79e^{-07}$	0.62
S. cerevisiae	8	expression-based, degree-based	$6.66e^{-26}$	2.55
S. cerevisiae	8	expression-based, random attachment	$1.03e^{-08}$	0.71
S. cerevisiae	8	degree-based, random attachment	$7.21e^{-01}$	0.04
S. cerevisiae	16	expression-based, degree-based	$2.03e^{-25}$	2.47
S. cerevisiae	16	expression-based, random attachment	$2.20e^{-04}$	0.40
S. cerevisiae	16	degree-based, random attachment	$6.84e^{-10}$	0.80
E. coli	4	expression-based, degree-based	$5.68e^{-27}$	2.73
E. coli	4	expression-based, random attachment	$4.52e^{-33}$	4.00
E. coli	4	degree-based, random attachment	$2.46e^{-29}$	3.17
E. coli	8	expression-based, degree-based	$2.93e^{-23}$	2.15
E. coli	8	expression-based, random attachment	$6.94e^{-30}$	3.28
E. coli	8	degree-based, random attachment	$2.83e^{-06}$	0.54
E. coli	16	expression-based, degree-based	$2.34e^{-33}$	4.07
E. coli	16	expression-based, random attachment	$1.81e^{-15}$	1.26
E. coli	16	degree-based, random attachment	$1.53e^{-19}$	1.68
H. sapiens	4	expression-based, degree-based	$4.75e^{-12}$	0.96
H. sapiens	4	expression-based, random attachment	$1.37e^{-22}$	2.06
H. sapiens	4	degree-based, random attachment	$6.30e^{-21}$	1.85
H. sapiens	8	expression-based, degree-based	$1.90e^{-15}$	1.26
H. sapiens	8	expression-based, random attachment	$1.39e^{-28}$	3.02
H. sapiens	8	degree-based, random attachment	$1.72e^{-16}$	1.36
H. sapiens	16	expression-based, degree-based	$1.12e^{-25}$	2.52
H. sapiens	16	expression-based, random attachment	$1.56e^{-31}$	3.64
H. sapiens	16	degree-based, random attachment	$2.74e^{-04}$	0.39

Table S1: ANCOVA results for pairwise prospective resilience slope comparisons in Figure 4. Almost all comparisons are significant under the Bonferroni-corrected significance threshold ($p < 0.0166$). Cohen's d was calculated from the ANCOVA's F-statistic for each comparison.

Supplementary Note S2

Supplemental tables. In Tables S1 and S2, we report the statistical significance values comparing the slopes of the various curves in Figure 4.

Supplementary Note S3

Prospective resilience across a variety of protein networks. Here we present additional findings to those in Figure 4 for DNA replication networks, mismatch repair networks, and protein export networks. These networks cover a variety of biological functions.

'DNA replication' is copying of the DNA molecule, a process containing many proteins.

Species	m	Pairwise comparison	p-value	Cohen's d
S. cerevisiae	4	expression-based, degree-based	$6.93e^{-01}$	0.04
S. cerevisiae	4	expression-based, random attachment	$6.65e^{-14}$	1.12
S. cerevisiae	4	degree-based, random attachment	$4.07e^{-12}$	0.97
S. cerevisiae	8	expression-based, degree-based	$4.49e^{-01}$	0.07
S. cerevisiae	8	expression-based, random attachment	$4.71e^{-25}$	2.42
S. cerevisiae	8	degree-based, random attachment	$4.07e^{-25}$	2.43
S. cerevisiae	16	expression-based, degree-based	$2.34e^{-01}$	0.12
S. cerevisiae	16	expression-based, random attachment	$6.31e^{-36}$	4.77
S. cerevisiae	16	degree-based, random attachment	$2.46e^{-33}$	4.07
E. coli	4	expression-based, degree-based	$7.63e^{-06}$	0.51
E. coli	4	expression-based, random attachment	$3.24e^{-04}$	0.39
E. coli	4	degree-based, random attachment	$4.13e^{-11}$	0.89
E. coli	8	expression-based, degree-based	$4.13e^{-06}$	0.52
E. coli	8	expression-based, random attachment	$1.92e^{-01}$	0.13
E. coli	8	degree-based, random attachment	$1.32e^{-05}$	0.49
E. coli	16	expression-based, degree-based	$8.36e^{-13}$	1.03
E. coli	16	expression-based, random attachment	$1.06e^{-04}$	0.42
E. coli	16	degree-based, random attachment	$1.74e^{-16}$	1.36
H. sapiens	4	expression-based, degree-based	$3.00e^{-24}$	2.30
H. sapiens	4	expression-based, random attachment	$6.39e^{-36}$	4.77
H. sapiens	4	degree-based, random attachment	$1.05e^{-32}$	3.91
H. sapiens	8	expression-based, degree-based	$4.65e^{-08}$	0.66
H. sapiens	8	expression-based, random attachment	$5.60e^{-30}$	3.30
H. sapiens	8	degree-based, random attachment	$1.49e^{-27}$	2.83
H. sapiens	16	expression-based, degree-based	$1.80e^{-10}$	0.84
H. sapiens	16	expression-based, random attachment	$4.43e^{-35}$	4.53
H. sapiens	16	degree-based, random attachment	$4.90e^{-33}$	3.99

Table S2: ANCOVA results for pairwise prospective modularity slope comparisons in Figure 5. Many comparisons are significant based on the Bonferroni-corrected significance threshold ($p < 0.0166$). Cohen's d was calculated from the ANCOVA's F-statistic for each comparison.

'Mismatch repair' is the pathway that describes corrections of DNA mismatches, generated during DNA replication. It is a process that prevents mutations from becoming permanent in dividing cells. The 'protein export' is the active transport of proteins from the cytoplasm to the exterior of the cell, generally speaking for eukaryotes. It is a process that transports newly synthesized proteins into or across the cell membrane, which consists of a translocation channel of a membrane protein complex.

DNA replication, mismatch repair and protein export are keywords for KEGG pathways [4], which we used to extract PPI networks. By running the protein IDs via SNAP [5] we extracted the curated interactions, which we used to model the biggest entity of each PPI network. The ribosome is a specific structural complex, whereas DNA replication, mismatch repair and protein export are a processes consisting of multiple protein complexes that are

not constantly compiled (as the ribosome). We thereafter extracted the largest component of the networks, which we used for our analyses.

We provide statistical descriptions of these different networks in Figures S3, S4, and S5 (subplots a-i in each), and we validate the main findings about network resilience from Figure 4 in subplots j-l of Figures S3, S4, and S5.

Supplementary References

[1] Pavel L. Krapivsky, Sidney Redner, and Francois Leyvraz. "Connectivity of growing random networks". In: *Physical Review Letters* 85.21 (2000), pp. 4629–4632. DOI: 10.1103/PhysRevLett.85.4629.

[2] Albert-László Barabási, Réka Albert, and Hawoong Jeong. "Mean-field theory for scale-free random networks". In: *Physica A* 272.1 (1999), pp. 173–187. DOI: 10.1016/S0378-4371(99)00291-5.

[3] Marinka Zitnik, Rok Sosič, Marcus W. Feldman, and Jure Leskovec. "Evolution of resilience in protein interactomes across the tree of life". In: *Proceedings of the National Academy of Sciences* 116.10 (2019), pp. 4426–4433. DOI: 10.1073/pnas.1818013116.

[4] Minoru Kanehisa, Yoko Sato, Miho Furumichi, Kanae Morishima, and Mao Tanabe. "New approach for understanding genome variations in KEGG". In: *Nucleic Acids Research* 47.D1 (2018), pp. D590–D595. DOI: 10.1093/nar/gky962.

[5] Jure Leskovec and Andrej Krevl. *SNAP Datasets: Stanford Large Network Dataset Collection*. http://snap.stanford.edu/tree-of-life/. June 2014.

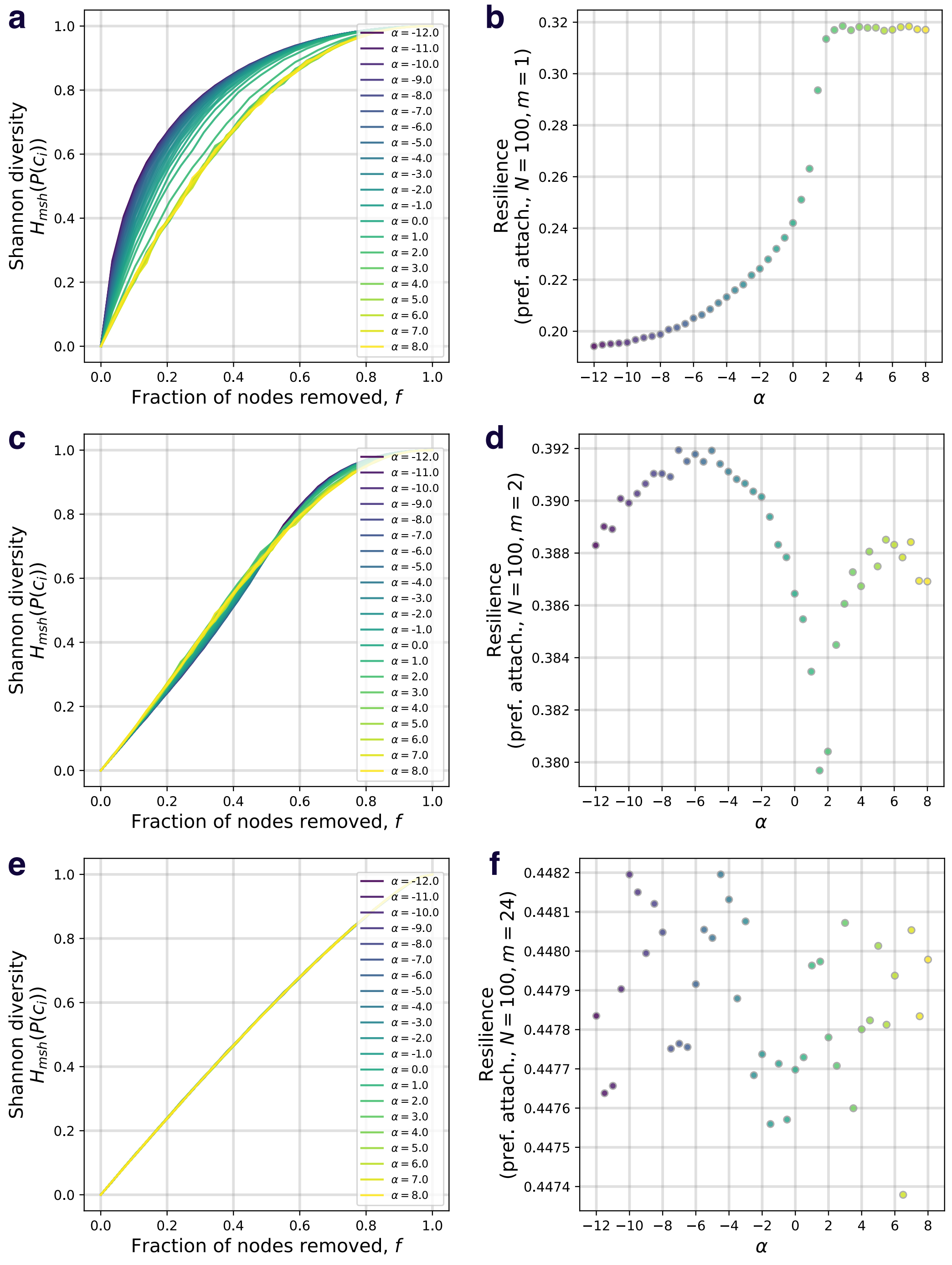

Figure S2: Preferential attachment and resilience. **(a, c, e)** An example of how Shannon Diversity changes as nodes are isolated from random attachment networks of 100 nodes for different values of α. The difference between **a**, **c**, and **e** are the number of dangling edges, m, a node has as it enters the network when the network is being generated. **(b, d, f)** The resilience values for the preferential attachment networks corresponding to each of the left hand plots.

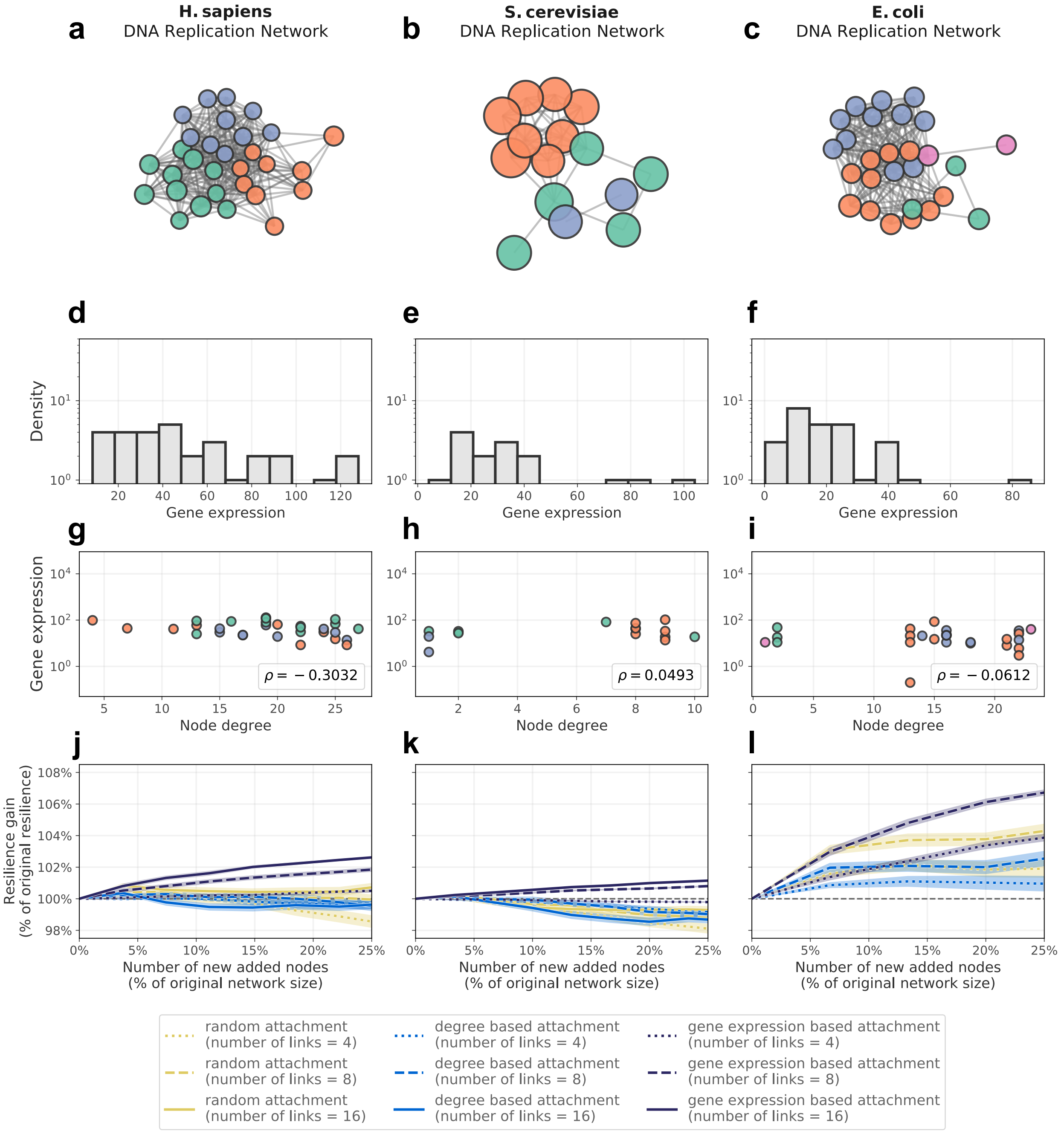

Figure S3: Prospective resilience of three DNA replication PPI networks. (a)-(i) Same network descriptions as Figure 2, but for the DNA replication networks. **(j)-(l)** The prospective resilience as these three networks gain new nodes, based on three node attachment mechanisms.

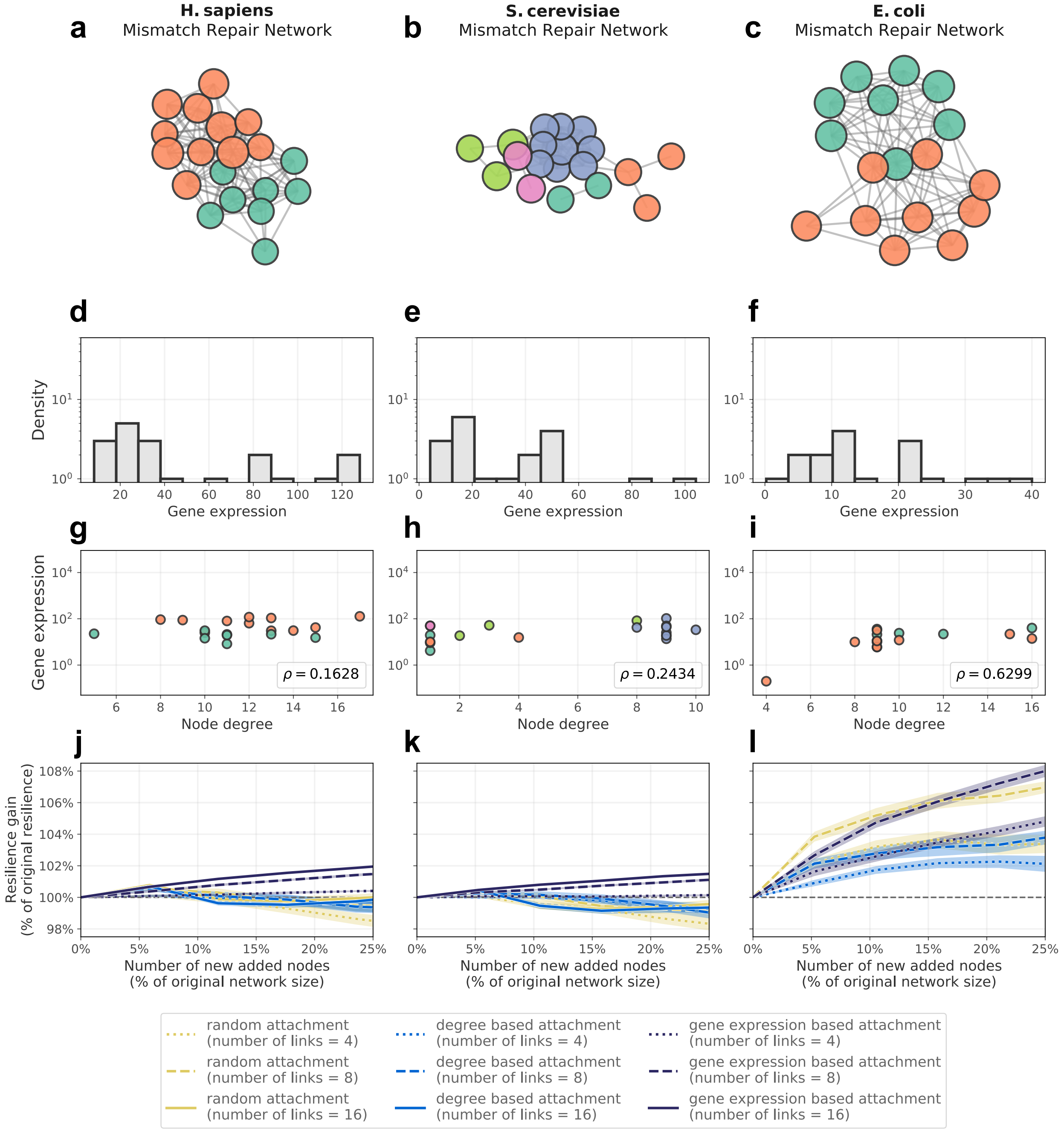

Figure S4: Prospective resilience of three mismatch repair PPI networks. (a)-(i) Same network descriptions as Figure 2, but for the mismatch repair networks. **(j)-(l)** The prospective resilience as these three networks gain new nodes, based on three node attachment mechanisms.

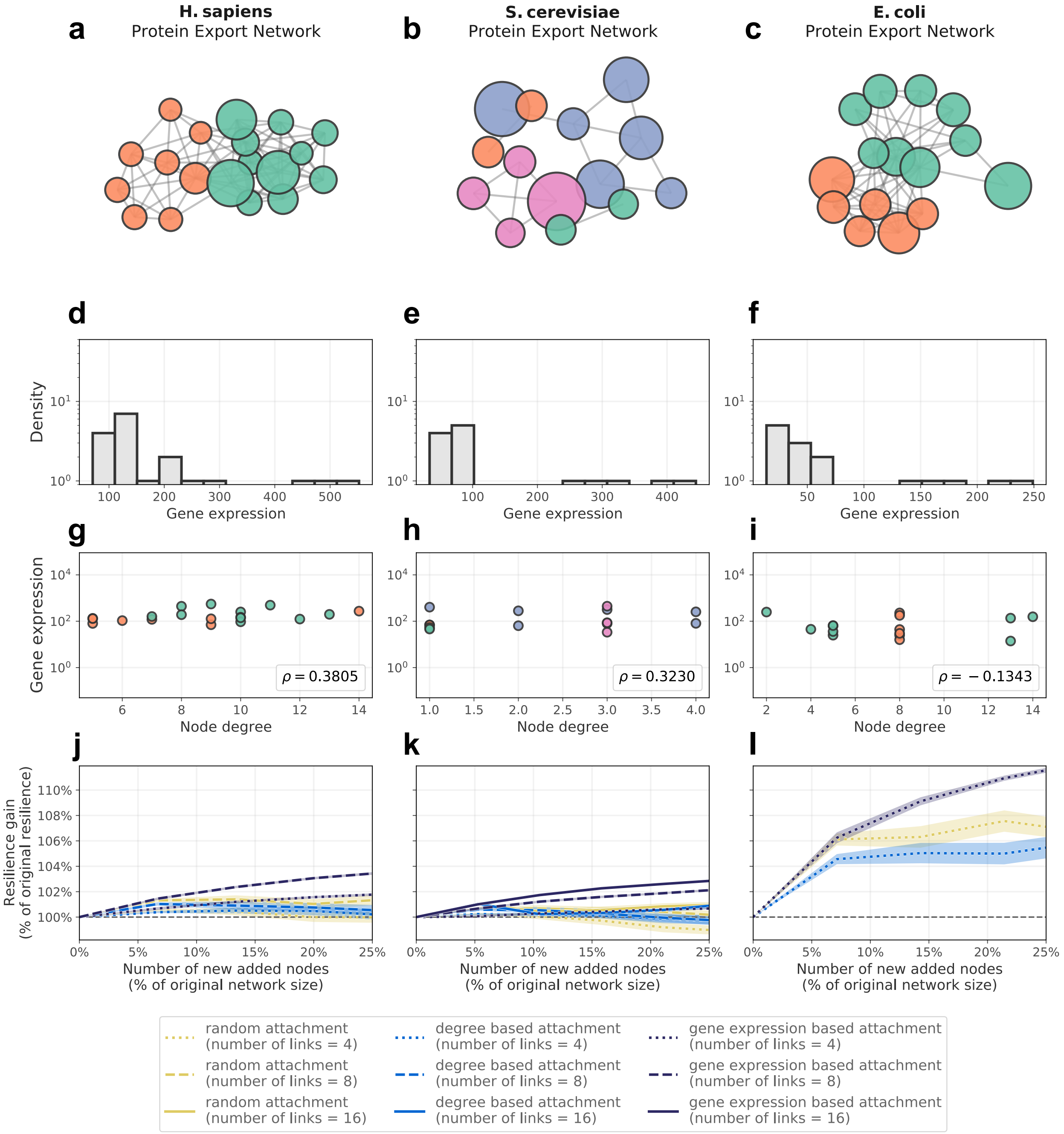

Figure S5: Prospective resilience of three protein export PPI networks. (a)-(i) Same network descriptions as Figure 2, but for the protein export networks. **(j)-(l)** The prospective resilience as these three networks gain new nodes, based on three node attachment mechanisms.
