# Supplementary material for: A computational exploration of resilience and evolvability of protein–protein interaction networks
Source: Commun Biol. 2021 Dec 2;4:1352. doi: 10.1038/s42003-021-02867-8 (PMC8639913; doi:10.1038/s42003-021-02867-8)
Supplement: Supplementary file 2 — Description of Additional Supplementary Files [file 42003_2021_2867_MOESM2_ESM.pdf]

Description of Additional Supplementary Files

File name: Supplementary Data 1.
Description: Includes data for reproducing Figures 4, 5, and 6. Network data for recreating Figure 2 is found in the accompanying repository (https://github.com/jkbren/presilience; doi: 10.5281/zenodo.5507368).
