# Supplementary material for: A computational exploration of resilience and evolvability of protein–protein interaction networks
Source: Commun Biol. 2021 Dec 2;4:1352. doi: 10.1038/s42003-021-02867-8 (PMC8639913; doi:10.1038/s42003-021-02867-8)
Supplement: Supplementary file 4 — Reporting summary [file 42003_2021_2867_MOESM4_ESM.pdf]

nature research

Corresponding author(s): Faysal Bibi

Last updated by author(s): Oct 4, 2021

Reporting Summary

Nature Research wishes to improve the reproducibility of the work that we publish. This form provides structure for consistency and transparency in reporting. For further information on Nature Research policies, see our Editorial Policies and the Editorial Policy Checklist.

Statistics

For all statistical analyses, confirm that the following items are present in the figure legend, table legend, main text, or Methods section.

n/a	Confirmed	
☐	☒	The exact sample size (*n*) for each experimental group/condition, given as a discrete number and unit of measurement
☒	☐	A statement on whether measurements were taken from distinct samples or whether the same sample was measured repeatedly
☐	☒	The statistical test(s) used AND whether they are one- or two-sided<br>*Only common tests should be described solely by name; describe more complex techniques in the Methods section.*
☐	☒	A description of all covariates tested
☐	☒	A description of any assumptions or corrections, such as tests of normality and adjustment for multiple comparisons
☐	☒	A full description of the statistical parameters including central tendency (e.g. means) or other basic estimates (e.g. regression coefficient) AND variation (e.g. standard deviation) or associated estimates of uncertainty (e.g. confidence intervals)
☐	☒	For null hypothesis testing, the test statistic (e.g. *F*, *t*, *r*) with confidence intervals, effect sizes, degrees of freedom and *P* value noted<br>*Give P values as exact values whenever suitable.*
☒	☐	For Bayesian analysis, information on the choice of priors and Markov chain Monte Carlo settings
☒	☐	For hierarchical and complex designs, identification of the appropriate level for tests and full reporting of outcomes
☒	☐	Estimates of effect sizes (e.g. Cohen's *d*, Pearson's *r*), indicating how they were calculated

Our web collection on statistics for biologists contains articles on many of the points above.

Software and code

Policy information about availability of computer code

Data collection	Artec Studio 13, iDav Landmark
Data analysis	R

For manuscripts utilizing custom algorithms or software that are central to the research but not yet described in published literature, software must be made available to editors and reviewers. We strongly encourage code deposition in a community repository (e.g. GitHub). See the Nature Research guidelines for submitting code & software for further information.

Data

Policy information about availability of data

All manuscripts must include a data availability statement. This statement should provide the following information, where applicable:

- Accession codes, unique identifiers, or web links for publicly available datasets
- A list of figures that have associated raw data
- A description of any restrictions on data availability

All 3D models, landmark data, and an R script for performing all analyses are available at DOI: 5 10.7479/499j-tv94 (an online repository of the Museum für Naturkunde).

Field-specific reporting

Please select the one below that is the best fit for your research. If you are not sure, read the appropriate sections before making your selection.

☐ Life sciences ☐ Behavioural & social sciences ☒ Ecological, evolutionary & environmental sciences

For a reference copy of the document with all sections, see nature.com/documents/nr-reporting-summary-flat.pdf

Ecological, evolutionary & environmental sciences study design

All studies must disclose on these points even when the disclosure is negative.

Study description	3D geometric morphometric analysis of bovid crania. All tests use functions from standard analytical packages in R.
Research sample	3D surface models of 123 bovid crania from the collections of the Museum für Naturkunde, Berlin, American Museum of Natural History, and Yale Peabody Museum
Sampling strategy	Crania chosen from existing datasets as well as by scanning specimens in collections in order to maximize species representation across bovids
Data collection	Scanning performed primarily by J.T. and student assistants. Landmarking performed by both authors.
Timing and spatial scale	Data collected between 2016-2021
Data exclusions	Not all data was used in all analyses. Only one specimen per species was randomly retained for most analyses in order to permit phylogenetically controlled testing.
Reproducibility	The analyses were repeated many times. Any differences in results among iterations of the analysis are noted in the text.
Randomization	Selection of one specimen per species was performed randomly with each iteration of the analysis R script. Specimens were assigned to dietary groups based on species accounts in the literature.
Blinding	NA

Did the study involve field work? ☐ Yes ☒ No

Reporting for specific materials, systems and methods

We require information from authors about some types of materials, experimental systems and methods used in many studies. Here, indicate whether each material, system or method listed is relevant to your study. If you are not sure if a list item applies to your research, read the appropriate section before selecting a response.

Materials & experimental systems

n/a	Involved in the study	
☒	☐	Antibodies
☒	☐	Eukaryotic cell lines
☒	☐	Palaeontology and archaeology
☐	☒	Animals and other organisms
☒	☐	Human research participants
☒	☐	Clinical data
☒	☐	Dual use research of concern

Methods

n/a	Involved in the study	
☒	☐	ChIP-seq
☒	☐	Flow cytometry
☒	☐	MRI-based neuroimaging

Animals and other organisms

Policy information about studies involving animals; ARRIVE guidelines recommended for reporting animal research

Laboratory animals	NA
Wild animals	NA
Field-collected samples	No field collection was involved. Studied crania were field or zoo-collected specimens and are housed at natural history museums.
Ethics oversight	NA.

Note that full information on the approval of the study protocol must also be provided in the manuscript.
